# Optimizing electromagnetic wave propagation in cylindrical structures with beam-plasma interactions: A mode-matching approach

**DOI:** 10.1371/journal.pone.0320307

**Published:** 2025-04-25

**Authors:** Shahana Rizvi, Muhammad Afzal

**Affiliations:** 1 Department of Mathematics, Capital University of Science and Technology, Islamabad, Pakistan; 2 Department of Mathematics and Natural Sciences, Center for Applied Mathematics and Bioinformatics, Gulf University for Science & Technology, Hawally, Kuwait; COMSATS University Islamabad, PAKISTAN

## Abstract

This study introduces a method for analyzing the propagation of electromagnetic waves in cylindrical structures with central chambers facilitating beam-plasma interactions, particularly relevant for slow-wave structures in backward wave oscillators. The boundary value problem, governed by the Helmholtz equation, is resolved using the mode-matching technique, yielding an exact solution. The analysis elucidates key phenomena, including reflection, transmission, orthogonality relations, and power flux variations with frequency and material properties. By examining the effects of plasma frequency and beam radius on phase velocity, group velocity, and interaction efficiency, the study provides insights into optimizing wave propagation and energy transfer. The results demonstrate that higher plasma frequencies and reduced beam radii enhance scattering characteristics, offering practical guidance for designing efficient electromagnetic devices.

## 1 Introduction

Cylindrical waveguides filled with cold plasma provide an innovative platform for manipulating electromagnetic fields, making them valuable in applications such as particle acceleration. As a result, the study of electromagnetic surface waves in plasma-filled waveguides has been extensively explored. In dense plasmas, nuclear reaction rates deviate significantly from their expected vacuum behavior due to many-body correlation effects and statistical phenomena unique to these systems. This has been extensively discussed in works like Ichimaru’s seminal review on nuclear fusion in dense plasmas [1]. The characteristics of electromagnetic waves play a crucial role in material sciences, as they significantly impact the design, performance, and discovery of advanced materials and technologies. Understanding how electromagnetic waves interact with different materials has paved the way for groundbreaking innovations, such as the development of novel aerogels with exceptional insulating and lightweight properties, metalenses capable of manipulating light at the nanoscale for improved imaging and optical devices, and advanced spectroscopy techniques used for precise material characterization and chemical analysis [2,3]. Furthermore, this knowledge has been instrumental in the creation of next-generation aviation equipment, where electromagnetic wave behavior is exploited for enhanced radar systems, stealth technology, and communication systems, contributing to safer and more efficient aerospace technologies [4,5]. In engineered systems, cold plasma is increasingly employed in cylindrical waveguides, which offer unparalleled opportunities for controlling electromagnetic wave propagation and power transmission. Waveguides partially or entirely filled with plasma enable new ways to study and exploit wave-plasma interactions. The propagation characteristics of surface electromagnetic waves in plasma waveguides, explored in studies such as Ivanov et al. [6], Ganguli et al. [7], and Ding et al. [8], demonstrate the potential of these systems to advance plasma-based technologies. Magnetized plasma waveguides, in particular, allow for the tailoring of dispersion properties to achieve specific operational goals, such as signal amplification or energy transfer [9–12]. Many microwave devices utilize cylindrical waveguides incorporating electron beams along their axes, where the interaction of the beam with electromagnetic waves enhances microwave signal amplification [13–17]. This beam-plasma interaction is fundamental in the design of microwave tubes, such as traveling wave tubes, widely used in satellite communications and radar systems [18].

Theoretical investigations, such as those by Nusinovich et al. [19], have analyzed space charge effects in plasma-filled traveling wave tubes, while Mishra et al. [20] examined the impact of plasma on the dispersive properties of these structures. In backward wave oscillators, the slow wave structure plays a critical role; for instance, Zhai et al. [21] demonstrated that introducing plasma into these structures modifies their dispersion characteristics. Beam-plasma systems are also integral to free electron devices, plasma-filled Cherenkov lasers, masers, high-power narrow-linewidth fiber laser and optical cavities used in enhanced light-nanomaterials interactions [22–24]. While experimental studies have advanced understanding of the dispersion properties, structural characteristics, and parameter dependencies of beam-plasma interactions [25–34], a comprehensive examination of electromagnetic scattering and power propagation within such systems remains limited.

This study addresses the existing gap by investigating the scattering of electromagnetic waves in a beam-plasma environment confined within a perfectly conducting cylindrical waveguide. The configuration features a central region where a beam-plasma, subject to a strong magnetic field, is situated between vacuum and magnetized plasma regions. The wave propagation starts in the left vacuum region, interacts with the central beam-plasma, and exits through the right plasma-filled region. To solve the scattering problem, the mode matching technique, an efficient and convergent semi-analytical method, is employed to derive exact expressions for the reflection and transmission coefficients. The structure of the article is as follows: Sect 2 formulates the boundary value problem for the scattering. In Sect 3, eigenfunction expansions are derived using the Helmholtz equation, and the mode matching technique is applied to solve for the unknown coefficients. Sect 4 rigorously establishes the power conservation principle. Sect 5 presents a detailed physical analysis that examines the impact of variations in plasma frequency, beam radius, and material properties. The paper concludes with a summary in Sect 6.

## 2 Problem formulation

**Fig 1:**
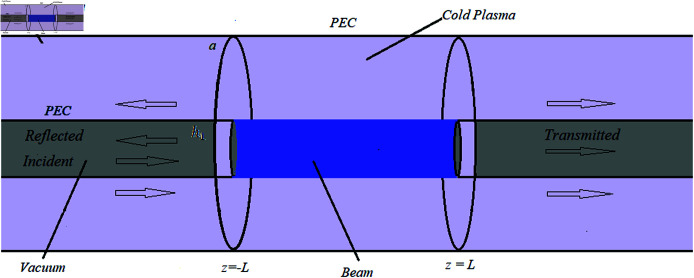
A cylindrical waveguide comprising of central chamber containing beam-plasma environment.

The infinite waveguide is bounded by a perfectly electric conducting boundary at r=a. The transmission of a transverse magnetic incident wave is analyzed as it propagates in the positive z-direction from the left conduit (z<−L) into the chamber, which is confined within the region |z|<L. The wave exits the chamber at the interface z=L and continues through the right side. This incident wave makes a zero angle with the z-axis and has unit amplitude. The regions |z|>L consist of vacuum and cold magnetized plasma, separated by a perfectly electric conducting wall at $r=h_{1}$. The beam within the central chamber is surrounded by cold magnetized plasma at $r=h_{1}$. Fig 1 provides a visual representation of the setup. The regions $|z|>L,0<r<h_{1}$, denoted as $R_{1}$ and $R_{4}$, contain vacuum, while the regions $|z|>L,h_{1}<r<a$ include plasma, represented as $R_{2}$ and $R_{5}$. The central region |z|<L is divided into sub-regions: one containing the beam and plasma within $0<r<h_{1}$ and another within $h_{1}<r<a$, labeled as $R_{3}$. The temporal variation $e^{-i\omega t}$, where ω denotes the angular frequency and i represents the imaginary unit $\left(i=\sqrt{-1}\right)$, is assumed and omitted throughout the article. It is important to note that the permittivity tensor ${\bar{\varepsilon }}_{j}$ (where j=p for cold plasma and j=b for plasma beam) is defined as

$${\bar{\varepsilon }}_{j}=\left[\begin{array}{ccc} \varepsilon_{1j} & -i\varepsilon_{2j} & 0\\ i\varepsilon_{2j} & \varepsilon_{1j} & 0\\ 0 & 0 & \varepsilon_{3j} \end{array}\right].$$

The components $\varepsilon_{1j}$ and $\varepsilon_{2j}$ are determined by analyzing the electromagnetic field properties in both cold plasma and the beam, and they are the same for both media. These components are expressed as

$$\varepsilon_{1j}=1-\frac{\omega_{p}^{2}}{\omega^{2}-\omega_{c}^{2}},\;\varepsilon_{2j}=\frac{\omega_{c}\omega_{p}^{2}}{\omega (\omega^{2}-\omega_{c}^{2})},$$

while the tensor component $\varepsilon_{3p}$ for plasma is given by $\varepsilon_{3p}=1-\frac{\omega_{p}^{2}}{\omega^{2}}$ [35], and for the beam, the component is expressed as $\varepsilon_{3b}=1-\frac{\omega_{p}^{2}}{\omega^{2}}-\frac{\omega_{b}^{2}}{\gamma^{3}{\left(\omega -k_{nz}v_{b}\right)}^{2}}$ [36]. In this context, $\omega_{p}$ and $\omega_{c}$ represent the frequencies corresponding to the plasma and cyclotron effects, respectively. The axial wave number is denoted as $k_{nz}=k_{0}\;+\;2n\pi$, where n=0,1,2,…. The relativistic factor is represented by γ, and $v_{b}$ denotes the beam velocity. It is important to note that the magnetic field $B_{0}$ is assumed to be very strong, so that $\left|\varepsilon_{2j}\right|$ is negligible, and $\varepsilon_{1j}=1$ [37].

### 2.1 Traveling wave formulation in regions ${\text{R}}_{1}$ and ${\text{R}}_{4}$

The expression for Ampere’s law in a vacuum, which relates the magnetic field B and the electric field E, is given by:

$$\begin{array}{ccc} \nabla \times B=-\frac{i\omega }{c^{2}}E, & & \end{array}$$ (1)

where ω is the angular frequency of the wave, c is the speed of light, and the terms represent the curl of the magnetic field and the electric field vector. The Helmholtz equation, which governs the propagation of electromagnetic waves in a waveguide, is expressed in terms of the longitudinal components of the electric and magnetic fields as follows:

$$\begin{array}{ccc} \left(\nabla^{2}+\frac{\omega^{2}}{c^{2}}\right)\left(\begin{array}{c} E_{z}\\ B_{z} \end{array}\right)=\left(\begin{array}{c} 0\\ 0 \end{array}\right), & & \end{array}$$ (2)

where $E_{z}$ and $B_{z}$ represent the longitudinal components of the electric and magnetic fields, respectively. This equation describes the wave behavior in the z-direction in terms of the longitudinal electric and magnetic fields. The transverse components of the electric and magnetic fields can be derived from the longitudinal components. The electric field components $E_{r}$ and $E_{\theta }$, as well as the magnetic field components $B_{r}$ and $B_{\theta }$, are given by:

$$E_{r}=\frac{is}{\eta^{2}}\frac{\partial E_{z}}{\partial r},\;E_{\theta }=0,$$

$$B_{r}=0,\;B_{\theta }=\frac{i\omega }{c^{2}\eta^{2}}\frac{\partial E_{z}}{\partial r},$$

where $\eta^{2}=\frac{\omega^{2}}{c^{2}}-s^{2}$ is the waveguide parameter, and s is the wavenumber in the radial direction. The electric potentials in the regions $R_{1}$ and $R_{4}$, which represent the vacuum regions, are denoted by $\phi_{1}$ and $\phi_{4}$, respectively. The boundary condition at the wall $r=h_{1}$ in the regions where |z|>L is given by:

$$\begin{array}{ccc} \frac{\partial \phi_{1}}{\partial r}(h_{1},z)=\frac{\partial \phi_{4}}{\partial r}(h_{1},z)=0, & & \end{array}$$ (3)

which represents the condition of zero radial electric field at the boundary, ensuring the field remains tangential at the conducting boundary. To solve the Helmholtz Eq (2), we apply the technique of separation of variables, assuming a solution of the form $\phi (r,z)=\Phi (r)e^{ik_{0}z}$, where $k_{0}$ is the propagation constant in the z-direction. This leads to the following eigenfunction expansions for the potentials $\phi_{1}$ and $\phi_{4}$:

$$\begin{array}{ccc} \phi_{1}(r,z)=e^{ik_{0}z}+\overset{\infty }{\underset{n=0}{\sum }}A_{n}e^{-is_{n}^{\left(I\right)}(z)}R_{1n}^{\left(I\right)}(r), & & \end{array}$$ (4)

$$\begin{array}{ccc} \phi_{4}(r,z)=\overset{\infty }{\underset{n=0}{\sum }}F_{n}e^{is_{n}^{\left(I\right)}(z)}R_{1n}^{\left(I\right)}(r), & & \end{array}$$ (5)

where $A_{n}$ and $F_{n}$ are the amplitudes of the waves in the regions $R_{1}$ and $R_{4}$, respectively. The wavenumbers $s_{n}^{\left(I\right)}$ are determined by the transverse boundary conditions and are given by:

$$s_{n}^{(I)2}=k_{0}^{2}-\eta_{n}^{2},$$

where $\eta_{n}$ is the n-th root of the characteristic equation, determined by the boundary conditions. In the regions $R_{1}$ and $R_{4}$, the eigenfunctions are expressed in terms of the Bessel function of the first kind:

$$R_{1n}^{\left(I\right)}(r)=AJ_{0}(\eta_{n}r),$$

where $J_{0}$ is the Bessel function of the first kind, and A is a normalization constant. These Bessel functions satisfy the usual orthogonality relation:

$$\int_{0}^{h_{1}}R_{1n}^{\left(I\right)}(r)R_{1m}^{\left(I\right)}(r)r\;dr=\delta_{mn}E_{1m}^{\left(I\right)},$$

where $\delta_{mn}$ is the Kronecker delta function and $E_{1n}^{\left(I\right)}$ is given by:

$$E_{1n}^{\left(I\right)}=\int_{0}^{h_{1}}R_{1n}^{(I)2}(r)r\;dr.$$

The values of $\eta_{n}$, where n=0,1,2,…, are the roots of the characteristic equation derived from the boundary condition in Eq (3). These roots are found by solving the equation:

$$\begin{array}{ccc} J_{0}^{\prime }(\eta_{n}h_{1})=0. & & \end{array}$$ (6)

This equation gives the allowed values of the wavenumbers $s_{n}^{\left(I\right)}$ that satisfy the boundary conditions for the cylindrical waveguide.

### 2.2 Traveling wave formulation in regions${\text{R}}_{2}$ and${\text{R}}_{5}$

The formulation of Ampere’s law for cold plasma is given by:

$$\begin{array}{ccc} \nabla \times B=-\frac{i\omega }{c^{2}}{\bar{\varepsilon }}_{p}E, & & \end{array}$$ (7)

where ${\bar{\varepsilon }}_{p}$ is the permittivity tensor for cold plasma, and E and B are the electric and magnetic fields, respectively. In the context of cold plasma wave propagation, the Helmholtz equation, when written in terms of the longitudinal components, is expressed as:

$$\begin{array}{ccc} \left(\nabla^{2}+T_{1}^{2}\right)\left(\begin{array}{c} E_{z}\\ B_{z} \end{array}\right)=\left(\begin{array}{c} 0\\ 0 \end{array}\right), & & \end{array}$$ (8)

where $T_{1}^{2}=\left(\frac{\omega^{2}}{c^{2}}-k_{nz}^{2}\right)\left(1-\frac{\omega_{p}^{2}}{\omega^{2}}\right)$, with $\omega_{p}$ being the plasma frequency and $k_{nz}$ the axial wavenumber. The transverse components of the fields can be derived as:

$$E_{r}=\frac{is}{\lambda^{2}}\frac{\partial E_{z}}{\partial r},\;E_{\theta }=0,$$

$$B_{r}=0,\;B_{\theta }=\frac{iT_{1}^{2}}{\omega \lambda^{2}}\frac{\partial E_{z}}{\partial r},$$

where $\lambda^{2}=T_{1}^{2}\;-\;s^{2}$, and s is the radial wavenumber. Since cold plasma is present throughout the waveguide, we now explore the eigenfunction expansions in the different regions. The field potentials in regions $R_{2}$ and $R_{5}$ are denoted as $\phi_{2}$ and $\phi_{5}$, respectively. The boundary conditions at the walls $r=h_{1}$ and r=a are given by:

$$\begin{array}{ccc} \frac{\partial \phi_{2}}{\partial r}(h_{1},z)=\frac{\partial \phi_{5}}{\partial r}(h_{1},z)=0, & & \end{array}$$ (9)

$$\begin{array}{ccc} \frac{\partial \phi_{2}}{\partial r}(a,z)=\frac{\partial \phi_{5}}{\partial r}(a,z)=0. & & \end{array}$$ (10)

Applying the separation of variables method, we obtain the following expansions for the field potentials:

$$\begin{array}{ccc} \phi_{2}(r,z)=e^{iT_{1}z}+\overset{\infty }{\underset{n=0}{\sum }}B_{n}e^{-is_{n}^{\left(II\right)}z}R_{1n}^{\left(II\right)}(r), & & \end{array}$$ (11)

$$\begin{array}{ccc} \phi_{5}(r,z)=\overset{\infty }{\underset{n=0}{\sum }}G_{n}e^{is_{n}^{\left(II\right)}z}R_{1n}^{\left(II\right)}(r), & & \end{array}$$ (12)

where $B_{n}$ and $G_{n}$ are the amplitudes of the n-th mode in regions $R_{2}$ and $R_{5}$, respectively. The wavenumbers for the modes in these regions are expressed as:

$$s_{n}^{(II)2}=T_{1}^{2}-\lambda_{n}^{2}.$$

The Bessel functions $R_{1n}^{\left(II\right)}(r)$ in these regions are given by:

$$R_{1n}^{\left(II\right)}(r)=\frac{B}{N_{0}^{\prime }(\lambda_{n}h_{1})}\left[N_{0}^{\prime }(\lambda_{n}h_{1})J_{0}(\lambda_{n}r)-J_{0}^{\prime }(\lambda_{n}h_{1})N_{0}(\lambda_{n}r)\right],$$

where $N_{0}$ and $N_{0}^{\prime }$ are the Bessel functions of the second kind and their derivatives, respectively.

These functions satisfy the orthogonality condition:

$$\int_{h_{1}}^{a}R_{1m}^{\left(II\right)}(r)R_{1n}^{\left(II\right)}(r)r\;dr=\delta_{mn}E_{1m}^{\left(II\right)},$$

where $\delta_{mn}$ is the Kronecker delta, and the integral $E_{1n}^{\left(II\right)}$ is defined as:

$$E_{1n}^{\left(II\right)}=\int_{h_{1}}^{a}R_{1n}^{(II)2}(r)r\;dr.$$

The characteristic equation derived from the boundary condition at r=a Eq (10) is:

$$\begin{array}{ccc} N_{0}^{\prime }(\lambda_{n}h_{1})J_{0}^{\prime }(\lambda_{n}a)-J_{0}^{\prime }(\lambda_{n}h_{1})N_{0}^{\prime }(\lambda_{n}a)=0, & & \end{array}$$ (13)

where $\lambda_{n}$ for n=0,1,2,… are the roots of this equation, which determines the allowed wavenumbers for the modes in these regions.

### 2.3 Traveling wave formulation in region${\text{R}}_{3}$

The region $R_{3}$ contains both cold plasma and the plasma beam. Before delving deeper, we first focus on formulating the wave propagation in the plasma beam. Ampere’s law, when applied to plasma beams, is given by:

$$\begin{array}{ccc} \nabla \times \text{B}=-\frac{i\omega }{c^{2}}{\bar{\varepsilon }}_{b}\cdot \text{E}. & & \end{array}$$ (14)

For the plasma beam, the Helmholtz equation takes the following form:

$$\begin{array}{ccc} \left(\nabla^{2}+T_{2}^{2}\right)\left(\begin{array}{c} E_{z}\\ B_{z} \end{array}\right)=\left(\begin{array}{c} 0\\ 0 \end{array}\right), & & \end{array}$$ (15)

where

$$T_{2}^{2}=\left(\frac{\omega^{2}}{c^{2}}-k_{nz}^{2}\right)\left(1-\frac{\omega_{p}^{2}}{\omega^{2}}-\frac{\omega_{b}^{2}}{\gamma^{3}{\left(\omega -k_{nz}v\right)}^{2}}\right).$$

The transverse components of the fields are expressed as:

$$E_{r}=\frac{is}{\chi^{2}}\frac{\partial E_{z}}{\partial r},\;E_{\theta }=0,$$

$$B_{r}=0,\;B_{\theta }=\frac{iT_{2}^{2}}{\omega \chi^{2}}\frac{\partial E_{z}}{\partial r},$$

where $\chi^{2}=T_{1}^{2}-s^{2}$. Boundary conditions at the walls $r=h_{1}$ and r=a within this central region are given as:

$$\begin{array}{ccc} \phi_{3}^{\left(I\right)}(h_{1},z)=\phi_{3}^{\left(II\right)}(h_{1},z), & & \end{array}$$ (16)

$$\begin{array}{ccc} \frac{\partial \phi_{3}^{\left(I\right)}}{\partial r}(h_{1},z)=\frac{\partial \phi_{3}^{\left(II\right)}}{\partial r}(h_{1},z), & & \end{array}$$ (17)

$$\begin{array}{ccc} \frac{\partial \phi_{3}^{\left(II\right)}}{\partial r}(a,z)=0. & & \end{array}$$ (18)

Here, $\phi_{3}$ represents the field potential in this region. By applying the separation of variables technique to Eqs (8) and (15), we obtain the eigenfunction expansion as follows:

$$\phi_{3}(r,z)=\{\begin{array}{cc} \overset{\infty }{\underset{n=0}{\sum }}\left(C_{n}e^{is_{n}z}+D_{n}e^{-is_{n}z}\right)R_{2n}^{\left(I\right)}(r),\; & 0<r<h_{1},\\ \overset{\infty }{\underset{n=0}{\sum }}\left(C_{n}e^{is_{n}z}+D_{n}e^{-is_{n}z}\right)R_{2n}^{\left(II\right)}(r),\; & h_{1}<r<a. \end{array}$$

Here, $C_{n}$ and $D_{n}$ represent the amplitudes of the n-th mode, and $s_{n}$ (where n=0,1,2,…) is the n-th mode wavenumber in this region. The Bessel functions in this region are given by:

$$R_{2n}^{\left(I\right)}(r)=CJ_{0}(\chi_{n}r),$$

$$R_{2n}^{\left(II\right)}(r)=\frac{D}{N_{0}^{\prime }(\gamma_{n}a)}\left\{N_{0}^{\prime }(\gamma_{n}a)J_{0}(\gamma_{n}r)-J_{0}^{\prime }(\gamma_{n}a)N_{0}(\gamma_{n}r)\right\}.$$

These functions satisfy the orthogonality relation:

$$\begin{array}{ccc} \int_{0}^{h_{1}}R_{2m}^{\left(I\right)}(r)R_{2n}^{\left(I\right)}(r)r\;dr+\int_{h_{1}}^{a}R_{2m}^{\left(II\right)}(r)R_{2n}^{\left(II\right)}(r)r\;dr=\delta_{mn}E_{m}, & & \end{array}$$ (19)

where

$$E_{n}=\int_{0}^{h_{1}}R_{2n}^{(I)2}(r)r\;dr+\int_{h_{1}}^{a}R_{2n}^{(II)2}(r)r\;dr.$$

The quantities $\chi_{n}^{2}=T_{1}^{2}\;-\;s_{n}^{2}$ and $\gamma_{n}^{2}=T_{2}^{2}\;-\;s_{n}^{2}$ (for n=0,1,2,…) are the roots of the equation derived from the boundary conditions in (16) and (17):

$$\begin{array}{ccc} \chi_{n}\left\{J_{0}^{\prime }(\chi_{n}h_{1})N_{0}^{\prime }(\gamma_{n}a)J_{0}(\gamma_{n}h_{1})-J_{0}^{\prime }(\chi_{n}h_{1})J_{0}^{\prime }(\gamma_{n}a)N_{0}(\gamma_{n}h_{1})\right\} & & \end{array}$$ (20)

$$=\gamma_{n}\left\{N_{0}^{\prime }(\gamma_{n}a)J_{0}^{\prime }(\gamma_{n}h_{1})J_{0}(\chi_{n}h_{1})-J_{0}^{\prime }(\gamma_{n}a)N_{0}^{\prime }(\gamma_{n}h_{1})J_{0}(\chi_{n}h_{1})\right\}.$$

## 3 Mode matching solution

In order to find the unknown coefficients appearing in the field potentials of different regions of this waveguide, we incorporate the matching conditions. The continuity of the electric and magnetic field potentials at the interfaces z=±L gives rise to the following matching conditions:

$$\begin{array}{ccc} \phi_{i}(r,\pm L)=\phi_{3}(r,\pm L),\;0\leq r\leq a, & & \end{array}$$ (21)

$$\begin{array}{ccc} \frac{\partial \phi_{i}}{\partial z}(r,\pm L)=\frac{\partial \phi_{3}}{\partial z}(r,\pm L),\;0\leq r\leq a, & & \end{array}$$ (22)

where i=1,2 at the interface z=−L, and at the interface z=L, i=4,5. Applying conditions (21) at the interface *z* = − *L*, we have

$$\begin{array}{ccc} 1+\overset{\infty }{\underset{n=0}{\sum }}A_{n}R_{1n}^{\left(I\right)}(r)=\overset{\infty }{\underset{n=0}{\sum }}\left(C_{n}e^{-is_{n}L}+D_{n}e^{is_{n}L}\right)R_{2n}^{\left(I\right)}(r), & & \end{array}$$ (23)

$$\begin{array}{ccc} 1+\overset{\infty }{\underset{n=0}{\sum }}B_{n}R_{1n}^{\left(II\right)}(r)=\overset{\infty }{\underset{n=0}{\sum }}\left(C_{n}e^{-is_{n}L}+D_{n}e^{is_{n}L}\right)R_{2n}^{\left(II\right)}(r), & & \end{array}$$ (24)

Multiplying both the sides of (23) by $R_{1m}^{\left(I\right)}(r)r$ and integrating from 0 to $h_{1},$ we get

$$\begin{array}{ccc} A_{m}=-\delta_{m0}+\frac{1}{E_{1m}^{\left(I\right)}}\overset{\infty }{\underset{n=0}{\sum }}\left(C_{n}e^{-is_{n}L}+D_{n}e^{is_{n}L}\right)P_{mn}, & & \end{array}$$ (25)

where

$$P_{mn}=\int_{0}^{h_{1}}R_{1m}^{\left(I\right)}(r)R_{2n}^{\left(I\right)}(r)rdr.$$

Solving in a similar manner, the Eq (24) produces

$$\begin{array}{ccc} B_{m}=-\delta_{m0}+\frac{1}{E_{1m}^{\left(II\right)}}\overset{\infty }{\underset{n=0}{\sum }}\left(C_{n}e^{-is_{n}L}+D_{n}e^{is_{n}L}\right)Q_{mn}, & & \end{array}$$ (26)

where

$$Q_{mn}=\int_{h_{1}}^{a}R_{1m}^{\left(II\right)}(r)R_{2n}^{\left(II\right)}(r)rdr.$$

Employing the matching conditions (21) at the interface *z* = *L* ,  yields

$$\begin{array}{ccc} \overset{\infty }{\underset{n=0}{\sum }}F_{n}R_{1n}^{\left(I\right)}(r)=\overset{\infty }{\underset{n=0}{\sum }}\left(C_{n}e^{is_{n}L}+D_{n}e^{-is_{n}L}\right)R_{2n}^{\left(I\right)}(r), & & \end{array}$$ (27)

$$\begin{array}{ccc} \overset{\infty }{\underset{n=0}{\sum }}G_{n}R_{1n}^{\left(II\right)}(r)=\overset{\infty }{\underset{n=0}{\sum }}\left(C_{n}e^{is_{n}L}+D_{n}e^{-is_{n}L}\right)R_{2n}^{\left(II\right)}(r), & & \end{array}$$ (28)

Applying the same procedure on (27) and (28), as was employed on (23) and (24), capitulates

$$\begin{array}{ccc} F_{m}=\frac{1}{E_{1m}^{\left(I\right)}}\overset{\infty }{\underset{n=0}{\sum }}\left(C_{n}e^{is_{n}L}+D_{n}e^{-is_{n}L}\right)P_{mn}, & & \end{array}$$ (29)

$$\begin{array}{ccc} G_{m}=\frac{1}{E_{1m}^{\left(II\right)}}\overset{\infty }{\underset{n=0}{\sum }}\left(C_{n}e^{is_{n}L}+D_{n}e^{-is_{n}L}\right)Q_{mn}. & & \end{array}$$ (30)

In the above-mentioned manner, by implementing the matching condition (22) at the interface *z* = − *L* ,  the following equations are derived,

$$\begin{array}{ccc} k_{0}-\overset{\infty }{\underset{n=0}{\sum }}A_{n}s_{n}^{\left(I\right)}R_{1n}^{\left(I\right)}(r)=\overset{\infty }{\underset{n=0}{\sum }}\left(C_{n}e^{-is_{n}L}-D_{n}e^{is_{n}L}\right)s_{n}R_{2n}^{\left(I\right)}(r), & & \end{array}$$ (31)

$$\begin{array}{ccc} T_{1}-\overset{\infty }{\underset{n=0}{\sum }}B_{n}s_{n}^{\left(II\right)}R_{1n}^{\left(II\right)}(r)=\overset{\infty }{\underset{n=0}{\sum }}\left(C_{n}e^{-is_{n}L}-D_{n}e^{is_{n}L}\right)s_{n}R_{2n}^{\left(II\right)}(r). & & \end{array}$$ (32)

Multiplying (31) by $R_{2m}^{\left(I\right)}(r)r$ and integrating from 0 to $h_{1}$, yields

$$\begin{array}{ccc} k_{0}P_{0m}-\overset{\infty }{\underset{n=0}{\sum }}A_{n}s_{n}^{\left(I\right)}P_{nm}=\overset{\infty }{\underset{n=0}{\sum }}\left(C_{n}e^{-is_{n}L}-D_{n}e^{is_{n}L}\right)s_{n}\int_{0}^{h_{1}}R_{2m}^{\left(I\right)}R_{2n}^{\left(I\right)}(r)rdr. & & \end{array}$$ (33)

Similarly, multiplying (32) with $R_{2m}^{\left(II\right)}(r)r$ and integrating from $h_{1}$ to *a* ,  the following equation is formed

$$\begin{array}{ccc} T_{1}Q_{0m}-\overset{\infty }{\underset{n=0}{\sum }}B_{n}s_{n}^{\left(II\right)}Q_{nm}=\overset{\infty }{\underset{n=0}{\sum }}\left(C_{n}e^{-is_{n}L}-D_{n}e^{is_{n}L}\right)s_{n}\int_{h_{1}}^{a}R_{2m}^{\left(II\right)}R_{2n}^{\left(II\right)}(r)rdr. & & \end{array}$$ (34)

Adding (33) and (34) and employing the derived orthogonality relation (19), we get

$$\begin{array}{cccc} C_{m}e^{-is_{m}L}-D_{m}e^{is_{m}L} & = & \frac{1}{s_{m}E_{m}}\left(k_{0}P_{0m}-\overset{\infty }{\underset{n=0}{\sum }}A_{n}s_{n}^{\left(I\right)}P_{nm}\right) & \\ & & +\frac{1}{s_{m}E_{m}}\left(T_{1}Q_{0m}-\overset{\infty }{\underset{n=0}{\sum }}B_{n}s_{n}^{\left(II\right)}Q_{nm}\right). \end{array}$$ (35)

Solving in a similar way, the deployment of condition (22) at the interface *z* = *L*, the following equations are formed,

$$\begin{array}{ccc} \overset{\infty }{\underset{n=0}{\sum }}F_{n}s_{n}^{\left(I\right)}R_{1n}^{\left(I\right)}(r)=\overset{\infty }{\underset{n=0}{\sum }}\left(C_{n}e^{is_{n}L}-D_{n}e^{-is_{n}L}\right)s_{n}R_{2n}^{\left(I\right)}(r), & & \end{array}$$ (36)

$$\begin{array}{ccc} \overset{\infty }{\underset{n=0}{\sum }}G_{n}s_{n}^{\left(II\right)}R_{1n}^{\left(II\right)}(r)=\overset{\infty }{\underset{n=0}{\sum }}\left(C_{n}e^{-is_{n}L}-D_{n}e^{is_{n}L}\right)s_{n}R_{2n}^{\left(II\right)}(r). & & \end{array}$$ (37)

Multiplication of (36) by $R_{2m}^{\left(I\right)}(r)r$ and integrating from 0 to $h_{1}$, yields

$$\begin{array}{ccc} \overset{\infty }{\underset{n=0}{\sum }}F_{n}s_{n}^{\left(I\right)}P_{nm}=\overset{\infty }{\underset{n=0}{\sum }}\left(C_{n}e^{is_{n}L}-D_{n}e^{-is_{n}L}\right)s_{n}\int_{0}^{h_{1}}R_{2m}^{\left(I\right)}R_{2n}^{\left(I\right)}(r)rdr. & & \end{array}$$ (38)

Following the aforementioned procedure forms the equation

$$\begin{array}{ccc} \overset{\infty }{\underset{n=0}{\sum }}G_{n}s_{n}^{\left(II\right)}Q_{nm}=\overset{\infty }{\underset{n=0}{\sum }}\left(C_{n}e^{is_{n}L}-D_{n}e^{-is_{n}L}\right)s_{n}\int_{h_{1}}^{a}R_{2m}^{\left(II\right)}R_{2n}^{\left(II\right)}(r)rdr. & & \end{array}$$ (39)

Adding (38) to (39) and employing the derived orthogonality relation (19), we get

$$\begin{array}{ccc} C_{m}e^{is_{m}L}-D_{m}e^{-is_{m}L}=\frac{1}{s_{m}E_{m}}\overset{\infty }{\underset{n=0}{\sum }}F_{n}s_{n}^{\left(I\right)}P_{nm}+\frac{1}{s_{m}E_{m}}\overset{\infty }{\underset{n=0}{\sum }}G_{n}s_{n}^{\left(II\right)}Q_{nm}. & & \end{array}$$ (40)

Adding (25) and (29) yields,

$$\begin{array}{ccc} \Psi_{1m}^{+}=-\delta_{m0}+\frac{2}{E_{1m}^{\left(I\right)}}\overset{\infty }{\underset{n=0}{\sum }}\Phi_{n}^{+}\cos(s_{n}L)P_{mn}. & & \end{array}$$ (41)

Similarly adding (26) to (30) renders

$$\begin{array}{ccc} \Psi_{2m}^{+}=-\delta_{m0}+\frac{2}{E_{1m}^{\left(II\right)}}\overset{\infty }{\underset{n=0}{\sum }}\Phi_{n}^{+}\cos(s_{n}L)Q_{mn}. & & \end{array}$$ (42)

Subtracting (29) from (25) and (30) from (26)

$$\begin{array}{ccc} \Psi_{1m}^{-}=-\delta_{m0}-\frac{2i}{E_{1m}^{\left(I\right)}}\overset{\infty }{\underset{n=0}{\sum }}\Phi_{n}^{-}\cos(s_{n}L)P_{mn}, & & \end{array}$$ (43)

$$\begin{array}{ccc} \Psi_{2m}^{-}=-\delta_{m0}-\frac{2i}{E_{1m}^{\left(II\right)}}\overset{\infty }{\underset{n=0}{\sum }}\Phi_{n}^{-}\cos(s_{n}L)Q_{mn}, & & \end{array}$$ (44)

where $\Psi_{1m}^{\pm }=A_{m}\pm F_{m},\Psi_{2m}^{\pm }=B_{m}\pm G_{m},$ and $\Phi_{m}^{\pm }=C_{m}\pm D_{m}.$ Subtracting (35) from (40), we obtain

Φn+=−k0P0m+T1Q0m2ismEm sin ⁡ (smL)+12ismEm sin ⁡ (smL)∑n=0∞Ψ1n+sn(I)Pnm+12ismEm sin ⁡ (smL)∑n=0∞Ψ2n+sn(II)Qnm. (45)

Adding (35) and (40), we get

Φn−=k0P0m+T1Q0m2smEm cos ⁡ (smL)−12smEm cos ⁡ (smL)∑n=0∞Ψ1n−sn(I)Pnm−12smEm cos ⁡ (smL)∑n=0∞Ψ2n−sn(II)Qnm. (46)

A system of infinite equations is represented by the Eqs (41)–(46), which involve the unknown coefficients $\left\{A_{n},B_{n},C_{n},D_{n},F_{n},G_{n}\right\}$ for *n* = 0 , 1 , 2 , … . This system is solved numerically after truncation and the results are presented and analyzed in the numerical section.

## 4 Energy flux

In order to assess the accuracy and convergence of the mode matching solution, it is essential to determine the energy flux. The Poynting vector plays a crucial role in this analysis, as it is employed to calculate the energy propagating through various sections of the waveguide. This approach allows for a detailed understanding of how energy flows within the system. In case of cylindrical setting, the Poynting vector is stated as [38],

$$\begin{array}{ccc} Power=\int_{R}\pi rRe\left\{E_{z}{\left(\frac{\partial E_{z}}{\partial z}\right)}^{*}\right\}dr, & & \end{array}$$ (47)

where (*) represents complex conjugate. The incident and reflected powers in the left duct *z* < − *L* ,  comprising of vacuum and plasma regions, are of the form

$$\begin{array}{ccc} P_{i}=P_{i}^{\left(I\right)}+P_{i}^{\left(II\right)}, & & \end{array}$$ (48)

$$\begin{array}{ccc} P_{r}=P_{r}^{\left(I\right)}+P_{r}^{\left(II\right)}. & & \end{array}$$ (49)

In the right duct *z* > *L* ,  the transmitted powers in the two regions can be described as

$$\begin{array}{ccc} P_{t}=P_{t}^{\left(I\right)}+P_{t}^{\left(II\right)}, & & \end{array}$$ (50)

Utilization of the Poynting vector, yields the incident $(P_{i}),$ reflected $\left(P_{r}\right)$ and transmitted $\left(P_{t}\right)$ powers as follows,

$$\begin{array}{ccc} P_{i}=\frac{\pi k_{0}h_{1}^{2}}{2}+\frac{\pi T_{1}(a^{2}-h_{1}^{2})}{2}, & & \end{array}$$ (51)

$$\begin{array}{ccc} P_{r}=-\pi Re\left(\overset{\infty }{\underset{n=0}{\sum }}|A_{n}{|}^{2}s_{n}^{\left(I\right)}E_{1n}^{\left(I\right)}\right)-\pi Re\left(\overset{\infty }{\underset{n=0}{\sum }}|B_{n}{|}^{2}s_{n}^{\left(II\right)}E_{1n}^{\left(II\right)}\right), & & \end{array}$$ (52)

$$\begin{array}{ccc} P_{t}=\pi Re\left(\overset{\infty }{\underset{n=0}{\sum }}|F_{n}{|}^{2}s_{n}^{\left(I\right)}E_{1n}^{\left(I\right)}\right)+\pi Re\left(\overset{\infty }{\underset{n=0}{\sum }}|G_{n}{|}^{2}s_{n}^{\left(II\right)}E_{1n}^{\left(II\right)}\right), & & \end{array}$$ (53)

The law of conservation of energy stated as,

$$P_{i}+P_{r}=P_{t}.$$

results in the following form,

$$\begin{array}{cccc} \frac{\pi k_{0}h_{1}^{2}}{2}+\frac{\pi T_{1}(a^{2}-h_{1}^{2})}{2}-\pi Re\left(\overset{\infty }{\underset{n=0}{\sum }}|A_{n}{|}^{2}s_{n}^{\left(I\right)}E_{1n}^{\left(I\right)}\right)-\pi Re\left(\overset{\infty }{\underset{n=0}{\sum }}|B_{n}{|}^{2}s_{n}^{\left(II\right)}E_{1n}^{\left(II\right)}\right) & & & \\ =\pi Re\left(\overset{\infty }{\underset{n=0}{\sum }}|F_{n}{|}^{2}s_{n}^{I}E_{1n}^{\left(I\right)}\right)+\pi Re\left(\overset{\infty }{\underset{n=0}{\sum }}|G_{n}{|}^{2}s_{n}^{\left(II\right)}E_{1n}^{\left(II\right)}\right). & & \end{array}$$ (54)

To reshape the Eq (54), the incident power $P_{i}$ is adjusted to a normalized value of 1 ,  such that

$$\begin{array}{ccc} 1=E_{1}+E_{2}+E_{3}+E_{4}, & & \end{array}$$ (55)

where

$$E_{1}=\frac{2}{K}Re\left(\overset{\infty }{\underset{n=0}{\sum }}|A_{n}{|}^{2}s_{n}^{\left(I\right)}E_{1n}^{\left(I\right)}\right),$$

$$E_{2}=\frac{2}{K}Re\left(\overset{\infty }{\underset{n=0}{\sum }}|B_{n}{|}^{2}s_{n}^{\left(II\right)}E_{1n}^{\left(II\right)}\right),$$

$$E_{3}=\frac{2}{K}Re\left(\overset{\infty }{\underset{n=0}{\sum }}|F_{n}{|}^{2}s_{n}^{\left(I\right)}E_{1n}^{\left(I\right)}\right),$$

$$E_{4}=\frac{2}{K}Re\left(\overset{\infty }{\underset{n=0}{\sum }}|G_{n}{|}^{2}s_{n}^{\left(II\right)}E_{1n}^{\left(II\right)}\right),$$

and

$$K=k_{0}h_{1}^{2}+T_{1}(a^{2}-h_{1}^{2}).$$

## 5 Numerical discussion

In this section, we present the outcomes of the numerical analysis conducted for the defined physical problem. The electric field potentials are depicted in the figures as below.:

$$\phi_{T}(r,z)=\{\begin{array}{cc} \phi_{1}(r,z),\;z<-L, & 0<r<h_{1},\\ \phi_{2}(r,z),\;z<-L, & h_{1}<r<a,\\ \phi_{3}(r,z),\;-L<z<L, & 0<r<a,\\ \phi_{4}(r,z),\;z>L, & 0<r<h_{1},\\ \phi_{5}(r,z),\;z>L, & h_{1}<r<a. \end{array}$$

The figures display the magnetic fields in their respective regions as follows

$$\phi_{Tz}(r,z)=\{\begin{array}{cc} \phi_{1z}(r,z),\;z<-L, & 0<r<h_{1},\\ \phi_{2z}(r,z),\;z<-L, & h_{1}<r<a,\\ \phi_{3z}(r,z),\;-L<z<L, & 0<r<a,\\ \phi_{4z}(r,z),\;z>L, & 0<r<h_{1},\\ \phi_{5z}(r,z),\;z>L, & h_{1}<r<a, \end{array}$$

where $\phi_{jz}=\frac{\partial \phi_{j}}{\partial z}\;;j=1,2,3,4,5.$

The physical parameters chosen are speed of light, $c=3\;\times \;10^{8}$ m/s, permittivity and permeability of free space, expressed respectively, as $\varepsilon_{0}=8.85\;\times \;10^{-12}$ F/m (Farad per meter), $\mu_{0}=4\pi \;\times \;10^{-7}$ N/A^2^ (Newtons per Ampere squared). The quantities $h_{1},a$ and *L* are the non–dimensional analogues of radii ${\bar{h}}_{1},\bar{a}$ and chamber length $\bar{L},$ respectively. To attain rigorous numerical results, the duct radii are set as $h_{1}=0.2$ cm and *a* = 0 . 4 cm. The beam velocity *v* is fixed at $0.134\times 10^{8}$ cm/second, while the frequencies are taken as $\omega =2.5\times 10^{9}$ radian/second, $\omega_{b}=2\;\times \;10^{9}$ radian/second and $\omega_{p}=10^{9}$ radian/second. The axial wavenumber is considered $k_{1z}=k_{0}+2\pi$. The chamber length *L* is set as 2 × *L* = 2 × 0 . 25 cm. The truncated system of equations is solved and the simulations are executed using the software Mathematica (version 12.1). The application of the mode matching technique has yielded a solution for the system outlined in Eqs (41)–(46), utilizing a truncation parameter denoted as *N*. Thus the unknown coefficients $\left\{A_{n},B_{n},C_{n},D_{n},F_{n},G_{n}\right\};\;n=0,1,2,\ldots ,99$ are determined. The solution is additionally utilized to confirm the accuracy of algebra, along with the principles of conservation and distribution of power.

**Fig 2:**
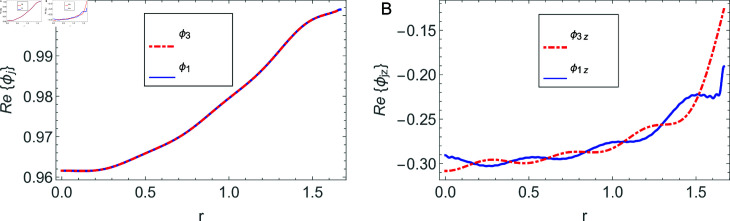
Real parts of the electric field and magnetic fields at the interface *z* = − *L* and $0\leq r\leq h_{1}.$

**Fig 3:**
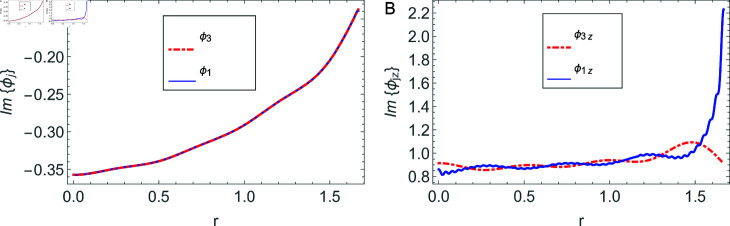
Imaginary parts of the electric field and magnetic fields at the interface *z* = − *L* and $0\leq r\leq h_{1}.$

**Fig 4:**
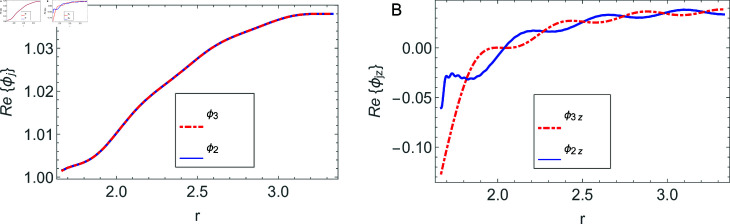
Real parts of the electric field and magnetic fields at the interface *z* = − *L* and $h_{1}\leq r\leq a.$

**Fig 5:**
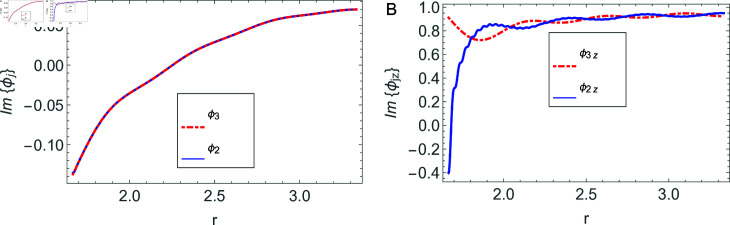
Imaginary parts of the electric field and magnetic fields at the interface *z* = − *L* and $h_{1}\leq r\leq a.$

**Fig 6:**
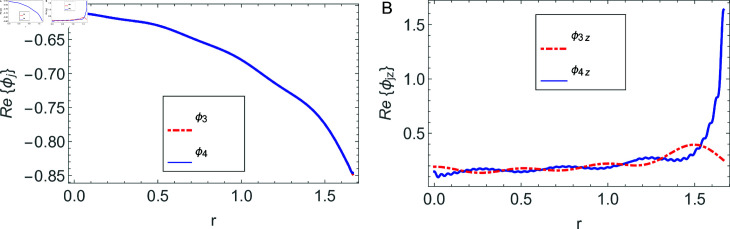
Real parts of the electric field and magnetic fields at the interface *z* = *L* and $0\leq r\leq h_{1}.$

**Fig 7:**
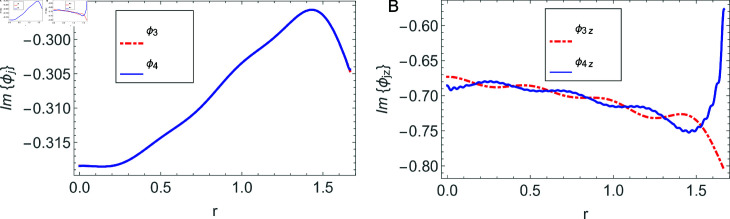
Imaginary parts of the electric field and magnetic fields at the interface *z* = *L* and $0\leq r\leq h_{1}.$

**Fig 8:**
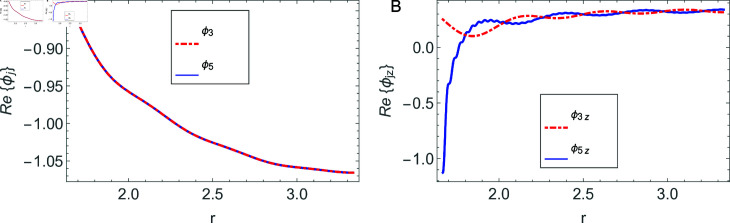
Real parts of the electric field and magnetic fields at the interface *z* = *L* and $h_{1}\leq r\leq a.$

**Fig 9:**
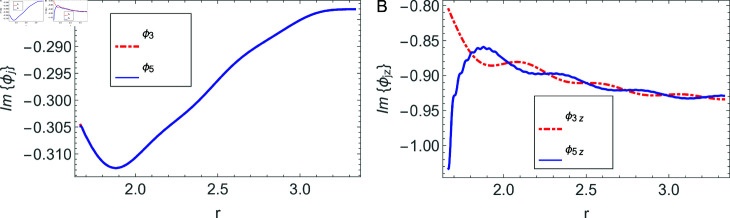
Imaginary parts of the electric field and magnetic fields at the interface *z* = *L* and $h_{1}\leq r\leq a.$

The accuracy of truncated solution is checked through reconstruction of the matching conditions at the two interfaces *z* = ± *L* and are displayed in Figs 2, 3, 4, 5, 6, 7, 8, 9. The real and imaginary parts of electric and magnetic field potentials completely coincide at the two interfaces in all mediums as is obvious from the figures.

The confirmation of the law of conservation of energy across different duct regions acts as an additional method to validate the precision of a truncated solution. The reflected powers in left duct  ( *z* < − *L* )  for regions $R_{1}$ and $R_{2}$ are denoted as $E_{1}$ and $E_{2}$, respectively while $E_{3}$ and $E_{4}$ represent the transmitted powers in the right duct  ( *z* > *L* ) . The sum of all powers $E_{t}$ is stated as

$$E_{t}=E_{1}+E_{2}+E_{3}+E_{4}.$$

**Fig 10:**
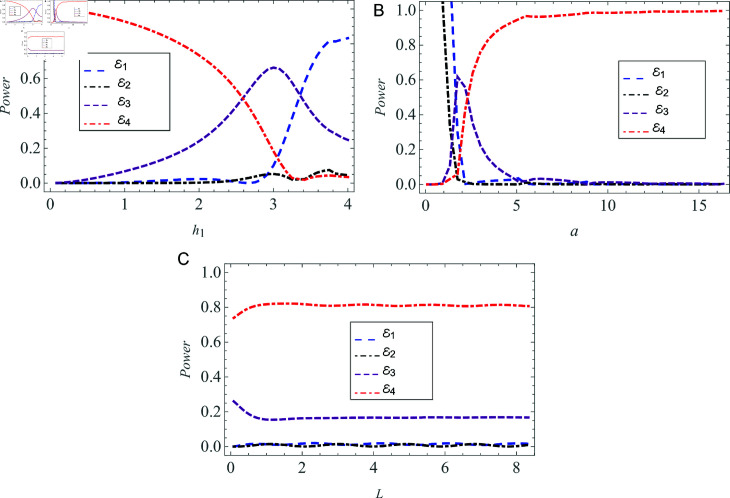
Power flux versus (A) beam radius $h_{1},(\text{B})$ plasma radius *a* ,  and  ( C )  chamber length *L* .

Fig 10 represents the effect of radii $h_{1},a$ and chamber length *L* on power flux. All parameters remain consistent with those selected for the matching conditions, with the sole difference being the parameter against which the power flux is plotted. In Fig 10A, the analysis of power flux is conducted in relation to the beam radius $h_{1}$, which ranges from 0 cm $<h_{1}<4$ cm. It has been noted that as $h_{1}$ increases, the reflection rises in the vacuum region, while both reflection and transmission effects diminish in the cold plasma region. Fig 10B indicates that transmission within the plasma is predominant, on the other hand, both transmission and reflection in the vacuum region approach zero as the plasma radius *a* increases. The permissible range for *a* is established as 0.01 cm  < *a* < 16 cm. The chamber length is considered as 0 < *L* < 8 cm for the plot of energy flux versus *L* .  Fig 10C reveals that an increase in the chamber length *L* leads to a greater transmission in plasma with minimal reflection. Additionally, extending *L* does not influence the energy flux in the vacuum, which consistently remains at zero.

**Fig 11:**
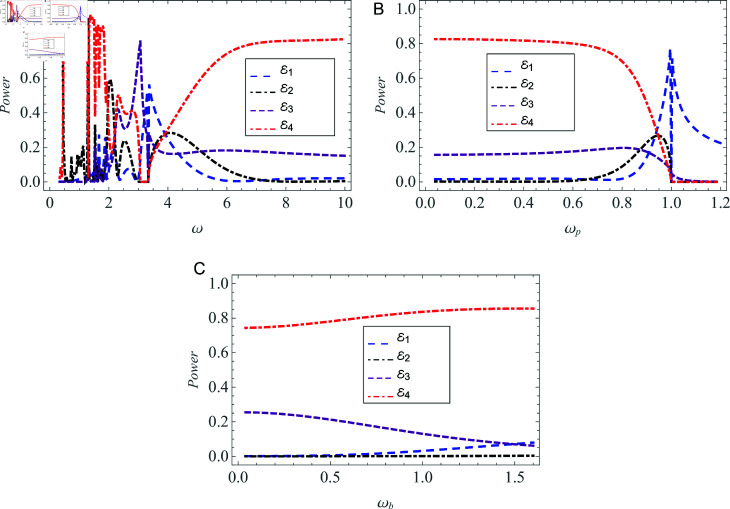
Power flux versus (A) angular frequency *ω* , ( B )  plasma frequency $\omega_{p},$ and  ( C )  beam frequency $\omega_{b}.$

Fig 11 illustrates the behavior of power flux in relation to angular, plasma, and beam frequencies. This plot examines the variations at these specific frequencies while keeping all other parameters constant. Based on Fig 11A, an increase in angular frequency *ω* leads to a substantial rise in the transmission of energy through plasma. In contrast, both transmission and reflection are nearly negligible in the vacuum. The specific range for *ω* is set up as 0 < *ω* ∕ *c* < 10 .  In Fig 11B, the increase in plasma frequency, $\omega_{p},$ indicates that reflection occurs within the vacuum, while both transmission through the plasma and vacuum are absent. Conversely, an increase in beam frequency, represented by $\omega_{b}$, suggests that transmission within the plasma medium increases while it diminishes in the vacuum, as illustrated in Fig 11C. The ranges of plasma and beam frequencies are set to be $0<\omega_{p}∕\omega <1.2$ and $0<\omega_{b}∕\omega <1.5.$

The peak values and sudden fluctuations observed in the graphs depicting scattering powers versus wave and plasma frequencies and plasma radius (obvious from Figs 10, 11) display the resonance behavior. In Fig 10B, a peak appears due to maximum value of transmission amplitude, at *a* = 1 . 75 cm. The reflection amplitudes in vacuum and plasma reach their maximum at *ω* ∕ *c* = 3 . 37 and *ω* ∕ *c* = 2 . 03 ,  respectively, implying peaks at these points. The transmission amplitude has a maximum in vacuum region at *ω* ∕ *c* = 3 . 07 .  Fig 11B shows a maximum value of reflection at $\omega_{p}∕\omega =1.$ At this point, the angular and plasma frequencies become equal. Beyond this threshold, while reflection is observed in the vacuum region, there is no transmission detected in either the plasma or the vacuum. This observation aligns with the principle that electromagnetic waves cannot propagate through plasma if the plasma frequency exceeds the wave or angular frequency.

**Fig 12:**
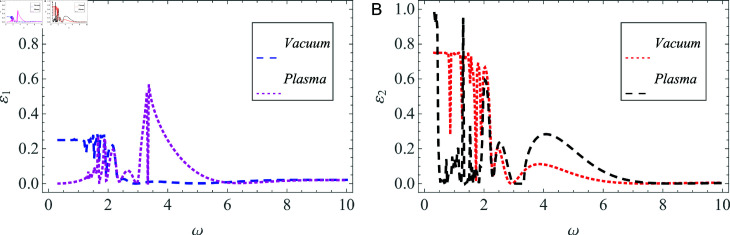
Comparison of reflected energy versus the angular frequency in (A) vacuum, and (B) plasma settings.

**Fig 13:**
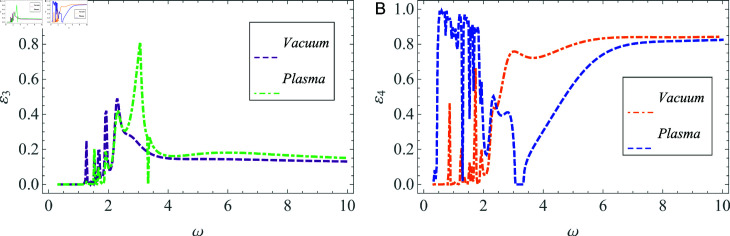
Comparison of transmitted energy versus the angular frequency in (A) vacuum, and (B) plasma settings.

The influence of change in material properties on power flow is also discussed, as indicated in Figs 12 and 13. The specified cylindrical waveguide is compared to a cylindrical structure that contains vacuum throughout all duct regions, except for the plasma beam, with a focus on energy flux to investigate this phenomenon. Fig 12A and 12B illustrate the reflected powers, whereas Fig 13A and 13B represent the transmitted powers in two different geometries characterized by unique material properties. It is evident that as the angular frequency *ω* increases, a significant rise in transmission is observed in the vacuum environment.

**Fig 14:**
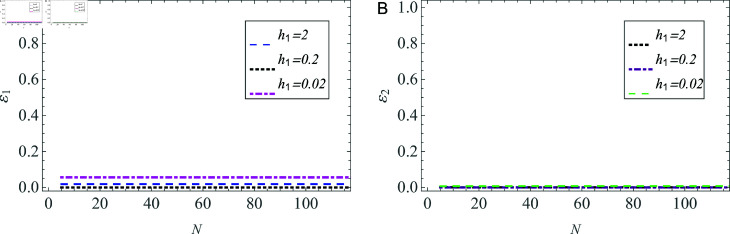
Reflected powers versus number of terms *N* in (A) vacuum, and (B) cold plasma, in different-sized waveguides.

**Fig 15:**
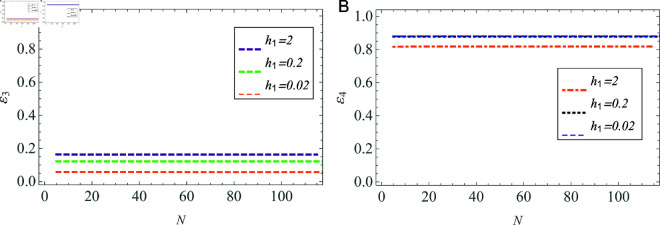
Transmitted powers versus number of terms *N* in (A) vacuum, and (B) cold plasma, in different-sized waveguides.

The convergence of the solution is examined also through the power conservation. The precision is verified to six decimal places. As indicated in Table 1, the impact of truncation becomes insignificant when *N* ≥ 105 .  Figs 14 and 15 further validate that the solution continues to exhibit convergence as the truncation number *N* increases across waveguides having varying widths of plasma beam. Specifically, Fig 14A and 14B illustrate the convergence of reflected powers in both vacuum and plasma regions, respectively. Furthermore, the convergence of transmitted powers is clearly illustrated in Fig 15A and 15B. Consequently, this system of infinite algebraic equations can be treated as finite. The beam radius $h_{1}$ is set to be 2 , 0 . 2 and 0 . 02 cm for these distinct waveguides, while the plasma radius is set as $a=3\times h_{1}$ (in cm).

**Table 1:** Conservation of power versus number of terms *N* .

Terms (*N*)	$E_{1}$	$E_{2}$	$E_{3}$	$E_{4}$	$E_{t}$
5	0.018271	0.000891	0.164164	0.816674	1
15	0.018611	0.000869	0.162672	0.817848	1
25	0.018578	0.000869	0.162813	0.817740	1
35	0.018569	0.000869	0.162851	0.817711	1
45	0.018565	0.000869	0.162867	0.817699	1
55	0.018564	0.000869	0.162875	0.817692	1
65	0.018562	0.000869	0.162880	0.817689	1
75	0.018562	0.000869	0.162881	0.817688	1
85	0.018561	0.000869	0.162885	0.817685	1
95	0.018561	0.000869	0.162886	0.817684	1
105	0.018561	0.000869	0.162887	0.817683	1
115	0.018561	0.000869	0.162887	0.817683	1

In context of cut-on modes, it is observed that only one cut-on mode exists in each region with increase in beam radius and angular frequency. The number of the cut-on modes increases in plasma regions in left and right duct as well as in central chamber with increase im plasma radius *a* ,  visible from Table 2.

**Table 2:** Cut-on modes versus plasma radius *a.*

Height (*a*)	$R_{1}$	$R_{2}$	$R_{3}$	$R_{4}$	$R_{5}$
3.33	1	1	1	1	1
5.17	1	1	2	1	1
5.33	1	2	2	1	2
8.75	1	2	3	1	2
8.92	1	3	3	1	3
12.25	1	3	4	1	3
15.75	1	3	5	1	3

## 6 Summary and conclusion

This study investigates the scattering behavior of transverse magnetic waves in a cylindrical waveguide with a beam-plasma region bounded by perfectly electric conducting walls, extending infinitely along the z-axis. The physical configuration considered involves vacuum-plasma regions in semi-bounded left and right ducts, separated by conducting walls, where the beam-plasma interaction plays a crucial role in high-power microwave pulse generation. A system of infinite algebraic equations was derived to analyze the reflection and transmission characteristics of the waveguide, with matching conditions ensuring the accuracy of the truncated solutions. Results demonstrate that the electric and magnetic fields are well-aligned at the two interfaces of the waveguide. An increase in the beam radius and plasma frequency resulted in a shift in the reflection behavior, leading to reflection occurring in the vacuum region, with no transmission observed through the plasma and vacuum interface. Additionally, as both plasma radius and angular frequency increased, transmission through the plasma improved significantly, while the vacuum remained unaffected. Furthermore, an increase in the beam frequency enhanced transmission through cold plasma. Notably, the length of the chamber did not influence the dominant transmission of electromagnetic waves through the plasma.

Power analysis conducted for cylindrical waveguides with varying beam radii and material properties corroborated the principle of power conservation. The convergence of solutions was observed for waveguides with varying plasma beam widths, suggesting that increasing plasma frequency and reducing beam radius can enhance nuclear target interaction. This results in a significant improvement in nuclear reaction rates and energy efficiency, with optimal values achieved at high plasma frequencies and small beam radii where particle velocity and beam density are maximized. This research paves the way for further studies on scaling cylindrical waveguides, incorporating grooves with plasma beams embedded in cold magnetized plasma, and investigating configurations bounded by dielectric materials. Future work could also explore structures with grooves, incorporating plasma beams between warm plasmas, to expand the potential for energy-efficient high-power microwave pulse generation and nuclear reaction optimization.
